# Estimating Postmortem Interval of Buried Pig Carcasses by Integrating Microbial Succession Patterns with Machine Learning Algorithms

**DOI:** 10.3390/microorganisms14010006

**Published:** 2025-12-19

**Authors:** Ting Yang, Xudong Chen, Qihua Xie, Jifeng Cai

**Affiliations:** Department of Forensic Science, Xiangya School of Basic Medical Sciences, Central South University, Changsha 410013, China; 236511098@csu.edu.cn (T.Y.); xdchen89@163.com (X.C.); xieqihua@csu.edu.cn (Q.X.)

**Keywords:** postmortem interval, buried carcasses, microbial succession, random forest algorithm, 16S rRNA gene sequencing

## Abstract

Microbial succession serves as a promising tool for estimating the postmortem interval (PMI). However, the patterns of microbial succession in burial scenarios require further exploration. This study established a pig carcass model, including buried and surface (control) groups, to investigate this. Using 16S ribosomal RNA (16S rRNA) gene sequencing, we analyzed microbial community changes and their differences across various decomposition stages. Results indicated that the decomposition rate of buried carcasses was slower than that of surface carcasses. Following the early decomposition stages, the alpha diversity of skin and underlying soil samples from buried carcasses decreased, a trend similar to that observed in the surface group. A significant shift in bacterial communities occurred in the buried group during abdominal rupture, mirroring the pattern in the surface group. At the phylum level, the relative abundance of Proteobacteria in the skin and soil of the buried group increased during later stages, consistent with the surface group. Furthermore, the buried and surface groups each possessed unique microbial taxa that responded to PMI changes. Using genus-level data, we identified feature taxa and constructed Random Forest models for PMI estimation. In the buried group, the mean absolute error (MAE) was 5.47 days for skin and 4.91 days for soil, while in the surface group, it was 5.59 days for skin and 5.30 days for soil. Although the model’s generalizability is currently limited by the sample size, the results demonstrate the predictability of microbial succession across different environmental contexts, underscoring its potential as a tool for PMI estimation in buried remains.

## 1. Introduction

The postmortem interval (PMI), defined as the time elapsed between death and the discovery of the corpse, plays a crucial role in forensic death investigations by defining the scope of investigations and verifying alibis [1]. Traditional PMI estimation methods include cadaveric temperature analysis [2], biochemical analysis of biofluids [3], analysis of macromolecular degradation patterns [4], and evaluation of developmental data from necrophagous insects [5]. These methods each have their own advantages but also have limitations. For example, methods based on body temperature are limited to early PMI (<72 h), while the entomological method is limited by species-specific colonization delays and relies on the presence of insects at the death scene. Moreover, cadaver decomposition involves complex interactions among autolytic enzymes, microbial communities, and environmental factors [6], increasing uncertainties in PMI estimation. Thus, forensic scientists are actively exploring other methods for estimating PMI.

In recent years, the emergence of forensic microbiology has offered a new research avenue for PMI estimation. The human microbiome shows distinct temporal changes during cadaver decomposition [7,8]. Endogenous microbial communities from living tissues and exogenous environmental microbial communities jointly serve as biological drivers of decomposition processes. The former initiates putrefaction by breaking through host immune barriers, and the latter speeds up the bioconversion of organic substances [9,10]. High-throughput sequencing allows for precise characterization of microbial community dynamics [11], thereby facilitating the development of microbial clocks. Notably, Metcalf et al. [12] developed a murine decomposition model with a prediction accuracy of ±3 days, and Johnson et al. [13] achieved a prediction accuracy of ±2 days through microbiome analysis of human cadaver skin. These breakthroughs highlight the translational potential of microbial biomarkers in forensic investigations.

The rupture of cadaveric tissues promotes the dispersal of internal microorganisms, thereby changing the structure of soil microbial communities in the surrounding environment. Organic decomposition fluids released in the process, such as amino acids and lipid metabolites, directly affect soil microbial communities. Cobaugh et al. [14] observed that although the total bacterial abundance remained relatively stable during decomposition, significant changes occurred in both soil function and microbial community structure. Weiss et al. [15] further showed that decomposition duration has a significant impact on soil microbial community composition, while cadaveric mass has no statistically significant effect. Considering the strong correlation between soil microbial dynamics and specific decomposition stages, soil microbial communities have been incorporated into predictive models for PMI estimation [16,17], highlighting their growing evidentiary value in forensic investigations.

Critically, current research mainly focuses on cadavers exposed on the terrestrial surface [18,19], resulting in knowledge gaps regarding the microecosystems of buried cadavers and related soil. Although Zhang et al. [20] found significant linear relationships between similarities of the microbial communities and postmortem intervals in buried rat models, and Yang et al. [21] observed that there were significant differences in the microbial communities of buried pigs before and after abdominal rupture, the reproducibility of microbial succession patterns, the presence of microbial biomarkers across geographic regions, and ecological interactions in burial environments remain uncharacterized. In this study, we used porcine models (*n* = 3 experimental, *n* = 3 surface controls) with 16S ribosomal RNA (16S rRNA) gene sequencing of skin and soil microbiomes across 10 time points (0–35 days) to describe buried and surface microbial succession, identify PMI-associated taxa, and develop a Random Forest predictive model. This study aims to compare bacterial dynamics between buried and surface carcasses, identify key microbial biomarkers in burial environments, and evaluate the accuracy of Random Forest models for PMI estimation based on characteristic microbial taxa.

## 2. Materials and Methods

### 2.1. Experimental Design and Sample Collection

The study was approved by the IRB of the School of Basic Medical Science, Central South University (No: 2020-KT58). To explore bacterial community succession in buried carcasses, six 2–3-month-old domestic pigs (*Sus scrofa domesticus*) (8.95–11.35 kg) were used as animal models. These pigs were purchased from a local farm. Prior to the experiment, all pigs were housed with ad libitum access to water and a commercial feed diet formulated for growing pigs. The animals were acclimatized to these conditions and showed no signs of disease. Animals were randomly divided into buried (*n* = 3) and surface (*n* = 3) groups. The experiment was conducted from 30 September to 4 November 2023 at an outdoor research site in Changsha County (28.34° N, 113.21° E), Hunan Province, China. The experimental site was located at the edge of suburban farmland, with a mix of green and dried weeds on the ground. After anesthesia, all pigs were subjected to precise percussive stunning of the head region to ensure a humane and rapid death. In the buried group, carcasses were buried in 100 cm × 70 cm × 60 cm pits and covered with 30 cm of original soil using metal mesh cages to simulate burial conditions. Sampling was performed by temporarily lifting the cage to expose the carcass. In the surface group, carcasses were placed on the ground inside protective cages to prevent scavengers from eating them.

Sampling was conducted at 10 postmortem intervals (0, 2, 4, 6, 9, 13, 16, 20, 25, and 35 days). Skin samples: At each sampling time point, a sterile cotton swab was used to gently wipe a 10 × 10 cm^2^ area of skin on one side of each carcass’s torso for 60 s, with care taken not to repeat any previously sampled areas. Subsequently, the tip of each swab was cut off with sterile scissors and placed into a 1.5 mL microcentrifuge tube. Soil samples: Soil samples were collected from the 0–5 cm depth beneath carcasses using sterilized spoons. Operators wore disposable gloves and masks, which were changed between handling different carcasses. All samples were immediately stored at −20 °C. To characterize the soil environment at the experimental site, soil samples were collected again from the adjacent areas of the buried and surface sampling sites (unaffected by decomposition) after the experiment was completed. These samples were treated as references for determining the initial soil physicochemical properties. Ambient temperature and rainfall data were obtained from a meteorological website during the experimental period. Photographs were taken at each sampling time point to visually document the progression of decomposition.

### 2.2. DNA Extraction, PCR Amplification, and Sequencing

Total genomic DNA was extracted from 120 samples using the TGuide S96 Magnetic Soil/Stool DNA Kit (Tiangen Biotech (Beijing) Co., Ltd., Beijing, China) according to the manufacturer’s instructions. The quality and quantity of the extracted DNA were examined using electrophoresis on a 1.8% agarose gel, and DNA concentration and purity were determined using a NanoDrop 2000 UV-Vis spectrophotometer (Thermo Scientific, Wilmington, NC, USA). The hypervariable V3-V4 region of the bacterial 16S rRNA gene was amplified using primer pairs 338F: 5′-ACTCCTACGGGAGGCAGCA-3′ and 806R: 5′-GGACTACHVGGGTWTCTAAT-3′. Both the forward and reverse 16S primers were tailed with sample-specific Illumina index sequences to enable deep sequencing. The PCR was carried out in a total reaction volume of 10 μL, containing DNA template (5–50 ng), 0.3 μL of forward primer (10 μM), 0.3 μL of reverse primer (10 μM), 5 μL of KOD FX Neo Buffer, 2 μL of dNTPs (2 mM each), 0.2 μL of KOD FX Neo, and ddH_2_O to make up the total volume to 10 μL. Initial denaturation was performed at 95 °C for 5 min, followed by 20 cycles of denaturation at 95 °C for 30 s, annealing at 50 °C for 30 s, and extension at 72 °C for 40 s, and then a final extension step at 72 °C for 7 min. The amplified products were purified with the Omega DNA purification kit (Omega Inc., Norcross, GA, USA) and quantified using the Qsep-400 (BiOptic, Inc., New Taipei City, Taiwan, China). The amplicon library was paired-end sequenced (2 × 250 bp) on an Illumina NovaSeq 6000 (Beijing Biomarker Technologies Co., Ltd., Beijing, China).

### 2.3. Bioinformatics and Statistical Analysis

Raw reads were quality-filtered using Trimmomatic v0.33 (SLIDINGWINDOW:4:20, MINLEN:50), and primers were removed using Cutadapt 1.9.1. Denoising, merging, and chimera removal were performed using DADA2 within QIIME2 2020.6 to generate amplicon sequence variants (ASVs) [22]. ASVs with a total count below 2 across all samples were subsequently filtered using QIIME2′s feature-table filter-features plugin. Taxonomic annotation of the ASVs was performed using the Naive Bayes classifier in QIIME2 [23] with the SILVA database (release 138.1) [24] and a confidence threshold of 70%. All samples were rarefied to the minimum sequence count (34,395 reads per sample) to normalize sequencing depth heterogeneity. Alpha diversity (Chao1 and Shannon) and rarefaction curves were calculated in QIIME2. For alpha diversity indices, the Wilcoxon rank-sum test was used for comparisons between groups at the same PMI, and the Kruskal–Wallis test for comparisons across PMIs. Inter-group differences were assessed using Principal Coordinate Analysis (PCoA) based on Bray–Curtis distances and PERMANOVA with 999 permutations.

### 2.4. Developing PMI Estimation Models

The genus-level relative abundance of bacteria in relation to PMI was analyzed using the randomForest package in R (version 4.5.1). Samples were divided into an 80% training set and a 20% test set based on the principle of stratified sampling. On the training set, the Boruta algorithm was used for feature selection with 500 iterations (*p*-Value = 0.01, mcAdj = TRUE, maxRuns = 500, doTrace = 2). The selection criterion was whether a feature’s importance score was significantly higher than that of random shadow features, and only features identified as “confirmed” were retained. For the selected features, the caret package was employed to perform a grid search on the mtry parameter via 10-fold cross-validation. The model was trained with the following settings: trControl = trainControl(method = “cv”, number = 10, verboseIter = TRUE, search = “grid”) and tuneGrid = expand.grid(mtry = unique(round(seq(1, max_mtry)))). The mtry value corresponding to the minimum root mean square error (RMSE) was selected. With the optimal mtry parameter fixed, hyperparameter adjustment was conducted for the number of decision trees (ntree), testing values from 500 to 2000 in increments of 100. The ntree value associated with the minimum out-of-bag (OOB) error was chosen as the final hyperparameter. A random forest model was then constructed using these optimized hyperparameters to predict the PMI. The model’s performance was evaluated on both the training set and test set using the mean absolute error (MAE) and the coefficient of determination (R^2^).

## 3. Results

### 3.1. Changes in Decomposition

Temperature and rainfall can exert a certain influence on the decomposition process of cadavers. We recorded the temperature and rainfall during the experimental period for reference (Figure S1). The daily mean temperature ranged from 16.5 to 26.0 °C, and the average daily rainfall was 2.53 mm. The soil was characterized as a light brown loam containing a small amount of clay. The initial soil parameters of the burial and surface sites showed partial similarities and differences (Table S1). While the pH (Buried group: 5.76 ± 0.06; Surface group: 5.52 ± 0.18) and dry matter moisture content (Buried group: 0.47 ± 0.11%; Surface: 0.44 ± 0.09%) were similar, the soil organic matter content at the burial sites (19.70 ± 1.30 g/kg) was lower than that at the surface sites (31.07 ± 1.16 g/kg). To investigate the impact of burial on the decomposition process, we compared the progression between buried and surface carcasses (Figure S2). During the 35-day decomposition process, distinct differences were observed between buried and surface carcasses. In buried carcasses, marked abdominal bloating appeared on day 4, followed by extensive abdominal rupture on day 9. In the later stage, due to the decomposition of carcasses and soil coverage, the signs of buried carcasses were not easy to observe, but the presence of their soft tissues could still be found. In contrast, surface carcasses exhibited accelerated decomposition. Abdominal bloating became evident on day 2, abdominal rupture occurred on day 6, and remains with many bones appeared on day 20. Buried carcasses followed a similar decomposition process to those on the surface, but their decomposition rate was significantly slower.

### 3.2. Sequencing Results

During the 35-day experimental period, sequencing of 120 samples generated 9,468,177 paired-end reads. Following processing steps, including quality control and assembly, a total of 8,459,324 clean reads were obtained, with each sample yielding a minimum of 40,752 clean reads and an average of 70,494 clean reads. These sequences were clustered into 125,193 amplicon sequence variants (ASVs). The rarefaction curves for all samples reached plateaus (Figure S3), demonstrating adequate sequencing depth to capture the majority of microbial diversity.

### 3.3. Bacterial Community Diversity Analysis

**Figure 1:**
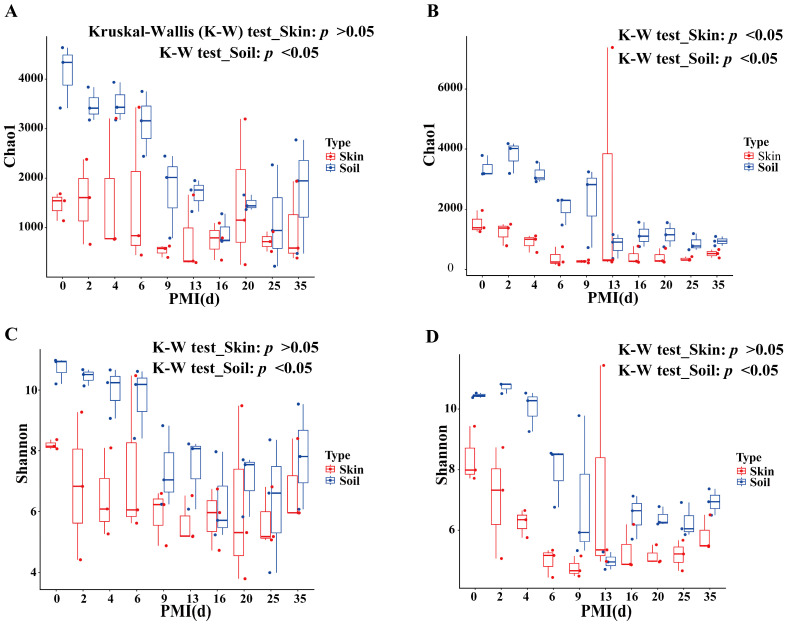
Changes in alpha diversity of skin and soil samples at different postmortem intervals. (**A**) Chao1 index in the buried group; (**B**) Chao1 index in the surface group; (**C**) Shannon index in the buried group; (**D**) Shannon index in the surface group.

The alpha diversity of samples was characterized using the Chao1 and Shannon indices (Figure 1). A general decreasing trend in the Chao1 index over time was observed in the skin and soil samples across different groups. The Shannon index also generally exhibited a declining trend during the initial stages of decomposition in all groups. After reaching the minimum value, it showed a slight rebound in the subsequent stages. However, it is noteworthy that even after the recovery period, the value of the Shannon index remained consistently lower than its level at the start of decomposition (day 0).

Statistical analysis using Kruskal–Wallis test revealed significant temporal differences in the alpha diversity of soil samples between the buried and surface groups at many time points (*p* < 0.05). In contrast, no significant temporal differences in alpha diversity were detected among the skin samples of the buried group (*p* > 0.05). For skin samples of the surface group, there was a significant difference in Chao1 index (*p* < 0.05), while no significant difference was observed in Shannon index. Furthermore, at the majority of time points, the alpha diversity of soil samples was higher than that of skin samples, irrespective of whether the samples were derived from the buried or surface group. However, when comparing groups at the same time points using Wilcoxon rank-sum test, no significant differences in alpha diversity were detected for skin and soil samples. As the sample size of each group was small, it might lead to insufficient statistical power to detect potential subtle differences.

**Figure 2:**
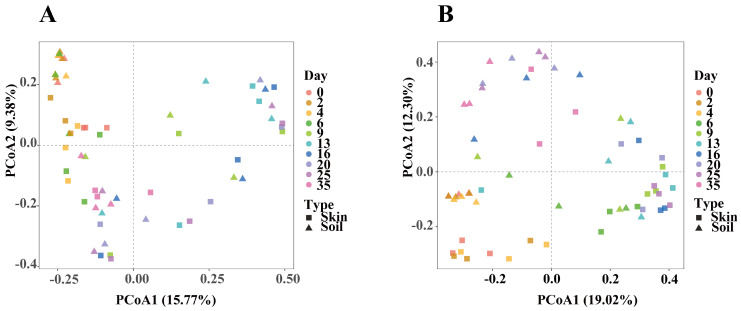
Principal Coordinates Analysis (PCoA) of skin and soil microbial communities based on Bray–Curtis distances. Filled colors correspond to PMI time points. Symbol shapes represent sample types. (**A**) Buried group; (**B**) Surface group.

To evaluate the differences in postmortem microbial communities between skin and soil samples from the buried and surface groups, we conducted a principal coordinates analysis (PCoA) based on Bray–Curtis distances (Figure 2). At the initial time point, there was a clear separation between skin and soil samples in both groups, with samples of the same type clustering together. As decomposition progressed, the microbial composition of all sample types showed a significant temporal shift. In particular, during the period when abdominal rupture occurred in the carcasses (days 6 to 9), the distance between samples increased significantly compared to the early stage of decomposition. Notably, in the late stage of decomposition, the distance between some samples of different types shortened, and the distinct separation observed in the early stage gradually disappeared.

To understand the effects of postmortem interval and sample type on microbial communities, a PERMANOVA analysis was performed, as shown in Table S2. Both PMI (Buried group: R^2^ = 0.26; Surface group: R^2^ = 0.27; both *p* < 0.001) and sample type (Buried group: R^2^ = 0.05; Surface group: R^2^ = 0.08; both *p* < 0.001) had significant effects on the microbial community structure. In terms of variance, the explanatory power of the PMI factor for community distribution was higher than that of the sample type, indicating that the time factor may have played a more important role in the successional pattern of microbial communities.

### 3.4. Temporal Changes in Bacterial Community Composition of Carcasses

**Figure 3:**
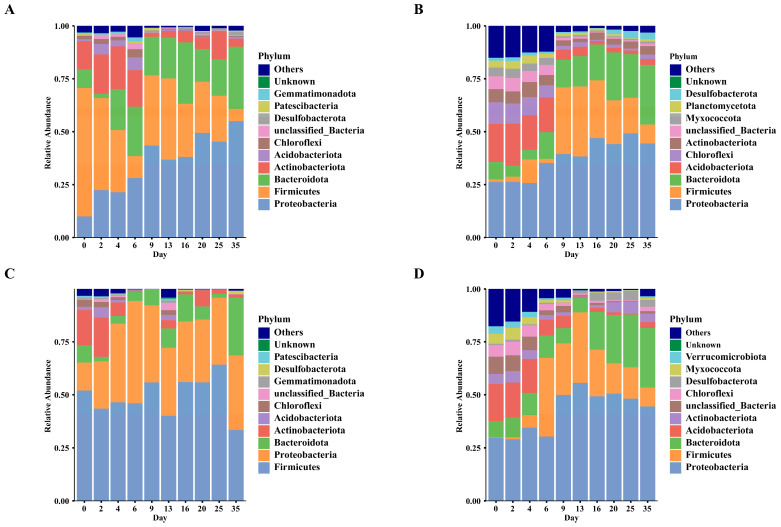
Daily changes in the relative abundance of bacteria at the phylum level across different sample types. Each unique color represents a distinct taxonomic phylum. (**A**) Skin samples of the buried group; (**B**) Soil samples of the buried group; (**C**) Skin samples of the surface group; (**D**) Soil samples of the surface group.

To identify the patterns of microbial changes with PMI across different sample types, we investigated the temporal dynamics of bacteria at both the phylum and genus levels. The results at the phylum level are shown in Figure 3. The dominant phyla in skin samples of the buried group were *Proteobacteria*, *Firmicutes*, *Bacteroidota*, and *Actinobacteriota*. In skin samples of the buried group, the relative abundances of *Proteobacteria* and *Bacteroidota* showed an upward trend from the early to the late decomposition stage, while the relative abundances of *Firmicutes* and *Acidobacteriota* in the late stage decreased compared with those in the early stage. The dominant phyla in skin samples of the surface group were *Firmicutes*, *Proteobacteria*, *Bacteroidota*, and *Actinobacteriota*. In the surface group, *Firmicutes* was the most abundant phylum, while the relative abundances of other phyla varied. In the late decomposition stage, the proportion of *Proteobacteria* increased significantly compared with the early stage, and the proportion of *Actinobacteriota* decreased significantly at the same time. In soil samples of both the buried and surface groups, the main components at the phylum level were *Proteobacteria*, *Firmicutes*, *Bacteroidota*, and *Acidobacteriota*. In the buried group, a significant shift in the bacterial community structure was observed on day 9. During the period of obvious abdominal rupture of the carcasses, it was characterized by a significant increase in the abundances of *Proteobacteria*, *Firmicutes*, and *Bacteroidota* compared with the earlier stage, while the abundance of *Actinobacteriota* decreased. Soil samples of the surface group showed a similar trend to those of the buried group. The abundance of *Proteobacteria* increased significantly after day 9 and tended to stabilize, whereas the abundance of *Acidobacteriota* decreased significantly.

**Figure 4:**
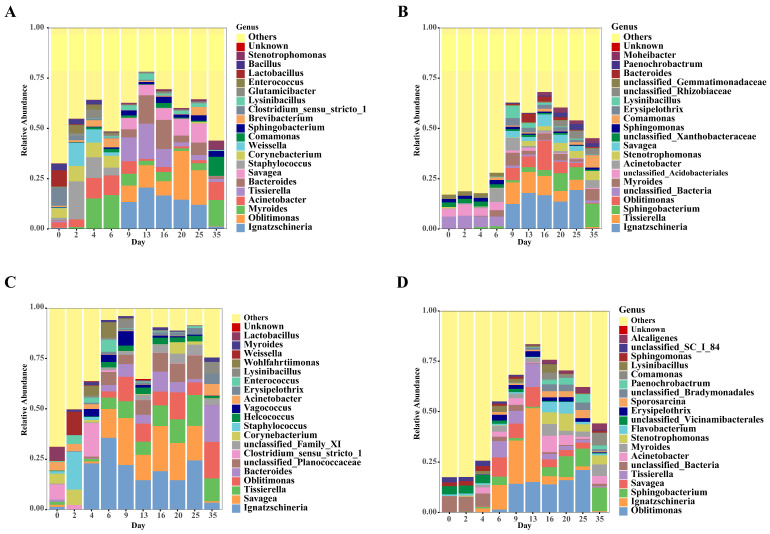
Daily changes in the relative abundance of bacteria at the genus level across different sample types. Each unique color represents a distinct genus. (**A**) Skin samples of the buried group; (**B**) Soil samples of the buried group; (**C**) Skin samples of the surface group; (**D**) Soil samples of the surface group.

As shown in Figure 4, we further observed the variation trend of microbial communities at the genus level. In the buried group’s skin samples, the early bacterial communities were dominated by *Acinetobacter*, *Myroides*, *Staphylococcus*, and *Corynebacterium*. While in the late stage, they shifted to be dominated by *Ignatzschineria*, *Oblitimonas*, *Savagea*, and *Tissierella*. For the surface group’s skin samples, the initial communities were dominated by *Clostridium_sensu_stricto_1*, *Corynebacterium*, and *Staphylococcus* and shifted to be dominated by *Ignatzschineria*, *Tissierella*, and *Oblitimonas* in the late stage. The buried group’s soil samples differed significantly between early and late stages and eventually became dominated by *Ignatzschineria*, *Savagea*, *Tissierella*, and *Oblitimonas*. The surface group’s soil samples showed a similar dominant pattern with *Oblitimonas* and *Ignatzschineria* as key taxa. These findings reveal the complexity and dynamics of bacterial community succession during decomposition, whose changes are affected by both environmental conditions and decomposition stages.

### 3.5. PMI Estimation Models Based on Skin and Soil Succession

**Figure 5:**
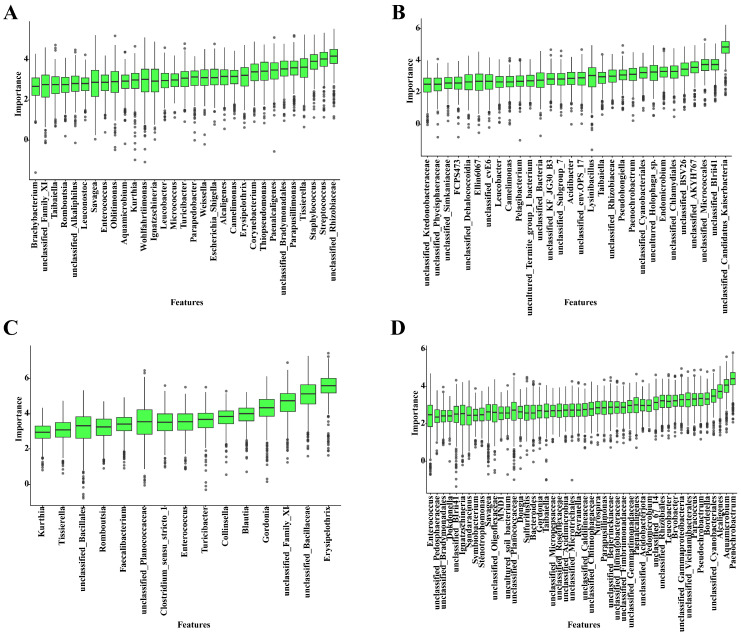
Feature selection results from the Boruta algorithm. The horizontal axis shows the selected bacterial genera, and the vertical axis represents feature importance score (Z-score). (**A**) Skin samples of the buried group; (**B**) Soil samples of the buried group; (**C**) Skin samples of the surface group; (**D**) Soil samples of the surface group.

To identify characteristic bacterial genera suitable for PMI prediction, we applied the Boruta algorithm to genus-level relative abundance data from the training set. As shown in Figure 5, we identified 31 and 30 important features for the buried group’s skin and soil samples, respectively. For the surface group, 15 and 48 important features were identified for skin and soil samples, respectively. These features were confirmed to be associated with PMI. Based on these selected features, we performed hyperparameter tuning for the Random Forest model, focusing on two key parameters: mtry and ntree. The specific model error under different hyperparameters is shown in Figure S4. The Random Forest model was established after hyperparameter optimization.

**Figure 6:**
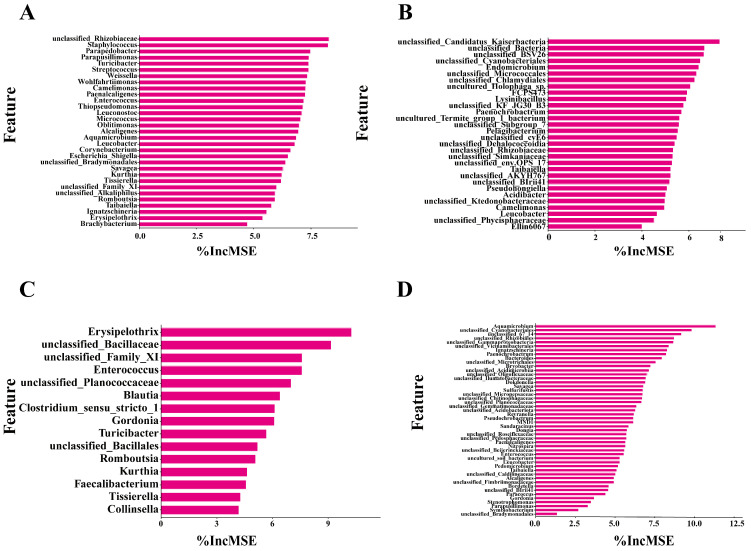
Bacterial genera feature importance rankings from the Random Forest model, evaluated by the percent increase in mean squared error (%IncMSE). (**A**) Skin samples of the buried group; (**B**) Soil samples of the buried group; (**C**) Skin samples of the surface group; (**D**) Soil samples of the surface group.

The Random Forest model ranked the importance of features, as shown in Figure 6. The most important features varied among groups. For skin samples, unclassified *Family_XI* and *Tissierella* were identified as important features in both the buried and surface groups. For soil samples, unclassified *cyanobacteriales* and *Taibaiella* were identified as important features in both groups. *Ignatzschineria* was identified as a biomarker for both skin samples in the buried group and soil samples in the surface group.

**Figure 7:**
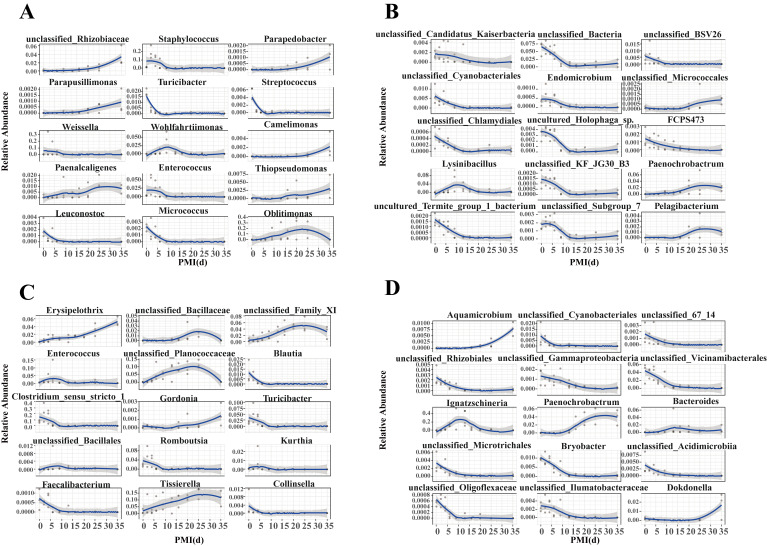
Changes in relative abundance of the top 15 contributing bacterial genera within the PMI estimation model. (**A**) Skin samples of the buried group; (**B**) Soil samples of the buried group; (**C**) Skin samples of the surface group; (**D**) Soil samples of the surface group.

To investigate the temporal dynamics of these key genera in relation to PMI, we analyzed the changes in relative abundance of the top 15 contributing features within the model. As shown in Figure 7, these genera exhibited successional patterns, with dominant taxa showing fluctuating trends and minor taxa displaying more sensitive, stage-specific responses. As decomposition progressed, the relative abundance of individual microbial genera was characterized by phases of increase, decline, or disappearance at specific postmortem intervals. For example, we observed that *Staphylococcus* decreased during days 2 to 9 and *Oblitimonas* increased during days 6 to 25 for skin samples in the buried group. Many bacteria with low relative abundance decreased during days 2 to 13 for soil samples in the buried group, such as unclassified *Cyanobacteriales* and uncultured *Holophaga_sp. Clostridium_sensu_stricto_1* decreased during days 2 to 6, and *Tissierella* increased during days 6 to 25 for skin samples in the surface group. We also observed that *Ignatzschineria* increased during days 6 to 9 and decreased during days 13 to 20 in soil samples of the surface group.

**Figure 8:**
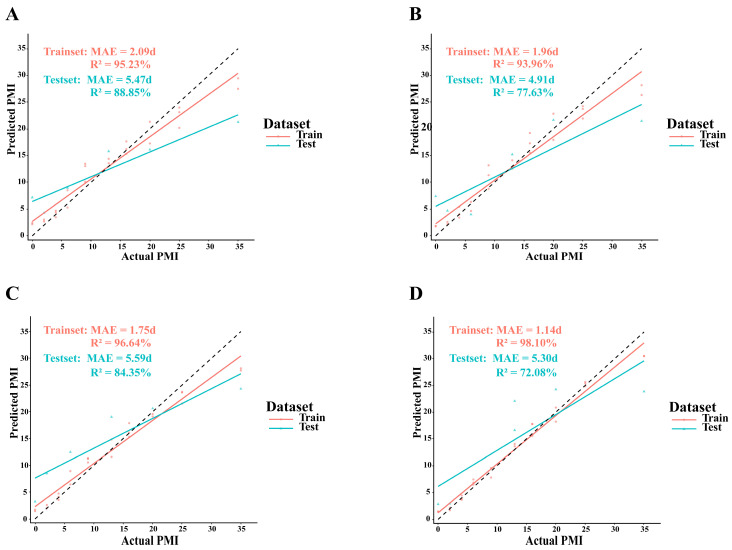
Comparison between predicted and actual PMI values for different sample types. Red points represent predicted values in training set. Blue points represent predicted values in test set. The black dashed line indicates perfect prediction. (**A**) Skin samples of the buried group; (**B**) Soil samples of the buried group; (**C**) Skin samples of the surface group; (**D**) Soil samples of the surface group.

We developed the Random Forest model to estimate the PMI based on selected characteristic bacterial genera. The predicted results are presented in Figure 8. For the buried group training set, the model achieved a Mean Absolute Error (MAE) of 2.09 days (R^2^ = 95.23%) for skin samples and 1.96 days (R^2^ = 93.96%) for soil samples. In the surface group training set, skin samples showed an MAE of 1.75 days (R^2^ = 96.64%), while soil samples had an MAE of 1.14 days (R^2^ = 98.10%). For the buried group test set, skin samples yielded an MAE of 5.47 days (R^2^ = 88.85%), and soil samples had an MAE of 4.91 days (R^2^ = 77.63%). In the surface group test set, skin samples showed an MAE of 5.59 days (R^2^ = 84.35%), while soil samples had an MAE of 5.30 days (R^2^ = 72.08%). Although all models performed well on the training set, their generalization ability on the test set was relatively limited, indicating a tendency for overfitting. This issue is primarily attributed to the limited sample size.

## 4. Discussion

Forensic microbiology has emerged as a promising tool for PMI estimation [25,26]. Current research on microbial-based estimation of PMI has predominantly focused on exposed cadavers in terrestrial surface environments [27,28]. However, accurate PMI estimation for buried cadavers is also important in forensic investigations. This study compared multiple aspects of buried and surface porcine carcasses, aiming to clarify the decomposition differences between the two environments and establish PMI prediction models.

The decomposition rate of buried carcasses was slower than that of surface carcasses. The delayed decomposition might be attributed to the combined effects of soil coverage, relatively lower temperature and limited oxygen availability. Soil coverage hindered the access of most insects and scavengers to carcasses [29]. Buried carcasses were in an environment with lower soil temperature. The exchange rate of oxygen and carbon dioxide in the soil possibly failed to meet the needs of aerobic microorganisms [30], which further inhibited decomposition.

Skin samples from buried and surface groups, as well as their corresponding soil samples, exhibited a decreasing trend in alpha diversity. The decline in alpha diversity during the decomposition process aligns with the findings of previous studies [31]. Notably, soil samples consistently demonstrated higher alpha diversity compared to skin samples across most sampling points. This difference may be attributed to the inherently richer carbon sources and nutritional substrates in soil ecosystems, which support more complex microbial communities.

This study evaluated the effects of PMI and sample type on microbial community dynamics. Results showed that both PMI and sample type significantly influenced microbial community structures (*p* < 0.001). All samples exhibited distinct temporal dynamics associated with PMI. PMI exerted a greater impact on microbial succession than sample type variation. This is consistent with the findings of Zhang et al. [20]. Microbial communities in skin and soil changed significantly before and after abdominal rupture. Li et al. [32] found that facultative anaerobes increased during abdominal distension but decreased after rupture. Cadaveric decomposition fluids significantly alter soil physicochemical parameters, triggering microbial community restructuring [33]. Notably, abdominal rupture was also a key stage for buried carcasses.

During the early decomposition phase, *Firmicutes*, *Actinobacteriota*, *Proteobacteria*, and *Bacteroidota* predominated in skin samples. In soil environments, the prokaryotic community during days 0–6 was dominated by *Proteobacteria*, *Bacteroidota*, and *Acidobacteriota*. These bacterial phyla were the dominant phyla in both burial and surface environments. Notably, *Proteobacteria* showed an increasing abundance trend in the later stage and became the most abundant phylum in soil samples. The finding is consistent with previous reports by Roesch et al. [34]. In the buried and surface groups, soil samples showed increasing trends for *Firmicutes* and *Bacteroidota* after abdominal rupture. These two phyla, recognized as dominant components of gut microbiota [35], played crucial roles in macromolecular and protein digestion. Postmortem intestinal bacteria likely initiate decomposition processes. Following carcass rupture, these bacteria translocate into the surrounding soil, explaining the observed increases in soil abundance.

At the genus level, the critical abdominal rupture phase (days 6–9) corresponded to maximal bacterial community shifts. In subsequent decomposition stages, the relative abundance of the genera *Ignatzschineria*, *Oblitimonas, Myroides, Acinetobacter, Tissierella*, and *Savagea* increased in skin samples. Notably, *Ignatzschineria*, *Tissierella, Savagea*, and *Oblitimonas* demonstrated similar increases in soil samples of both the buried and surface groups, suggesting their potential key roles in cross-environmental migration or metabolic adaptation during decomposition. *Ignatzschineria* was associated with necrophagous insects [36,37]. Its dominance in buried carcass bacterial communities and increase during decomposition suggest that necrophagous insects could either penetrate the soil or reproduce in the burial environment. Additionally, we opened the cage for buried carcasses. The action might attract insects to colonize and accelerate the rate of decomposition.

Machine learning methods (e.g., SVM, XGBoost, RF) can identify variation patterns in complex and diverse microbiome datasets and then use these patterns to achieve target prediction [38]. SVM strongly relies on kernel function and parameter tuning [39] and may struggle with the inherent complex non-linearity of microbiome data. XGBoost remains susceptible to overfitting on small datasets and requires more careful parameter tuning to achieve optimal performance [40]. In contrast, Random Forest exhibits rapid training speed, relatively straightforward tuning procedures, and robustness to noise, and it has been widely applied in forensic PMI estimation models [41]. The study by Belk et al. [42] demonstrated that cadaveric skin and cadaver-associated soil represent promising sampling sites for developing microbial tools to estimate PMI. PMI prediction models revealed distinct sets of signature genera across different environments and sample types. Despite performing feature selection and hyperparameter tuning, the model overfitted the training data due to small sample size and generalized poorly to the test set. Future studies should increase the sample size, consider the key event of abdominal rupture, and attempt to establish a stage-specific model.

Microbial technology offers objective, time-dependent data across all decomposition stages, particularly when traditional methods are no longer effective. However, microbial methods remain limited by high costs, reliance on specialized analysis, and sensitivity to environmental variables. Postmortem phenomena are straightforward for the early PMI estimation (<3 days) but highly environmentally sensitive [43]. Vitreous humor chemistry extends estimation to about 10 days but requires lab analysis [44]. Forensic entomology is key for long-term PMI estimation but relies on insect accessibility [45]. In comparison, microbial succession provides an insect-independent, objective microbial clock for the middle- and long-term PMI estimation. There is no single universally reliable method in PMI estimation. In the future, integrating microbial indicators with other estimating factors may further improve the accuracy of PMI estimation.

This study demonstrated the differences in microbial communities between the skin of pig carcasses and soil under buried and surface environments, as well as the potential applicability of microbial data in PMI estimation. Relevant studies have confirmed that human microbial changes also exhibit temporal patterns [46,47,48], which suggests that human microbial communities have value for PMI estimation. Due to differences in dietary patterns and gut microbial communities between pigs and humans, the results obtained from the pig models might not be suitable for human corpses. In addition, PMI estimation is subject to interference from multiple factors. Environmental variables (burial depth, soil texture, temperature, rainfall) can alter microbial community composition and thus impact the reliability of microbial indicators. It is important to note that this study was based on specific soil type, climatic conditions and animal model. These specific conditions might limit their extrapolation to other environments. Future validation under diverse conditions is necessary to develop applicable PMI estimation models.

## 5. Conclusions

This study investigated the dynamic changes in microbial communities in cadaveric skin and soil under both buried and surface environments, revealing the temporal patterns of microbial composition and abundance during the decomposition process. Potential PMI-related biomarkers were identified. For the buried group, the MAE was 5.47 days for skin samples (R^2^ = 88.85%) and 4.91 days for soil samples (R^2^ = 77.63%). For the surface group, the MAE was 5.59 days for skin samples (R^2^ = 84.35%) and 5.30 days for soil samples (R^2^ = 72.08%). Limited by the small sample size, the Random Forest model demonstrated insufficient generalizability, yet exhibited the potential of using microbial community data for PMI estimation. This research provides valuable insights into the role of microbial communities in PMI estimation under different environmental conditions.

## Data Availability

The original data presented in this study is publicly available on NCBI. The all-raw sequences are stored in the sequence read file (accession number: PRJNA1274674).

## Supplementary Materials

The following supporting information can be downloaded at https://www.mdpi.com/article/10.3390/microorganisms14010006/s1: Figure S1: Daily variations in ambient temperature and rainfall during the study period. Temperature data were obtained from https://www.weather.com.cn (accessed on 4 April 2025), and rainfall data were obtained from https://www.caomeikeyan.com (accessed on 15 August 2025); Figure S2: Morphological changes in buried and surface carcasses at different postmortem intervals. (A) Buried carcass; (B) Surface carcass. Figure S3: Rarefaction curves of skin and soil samples. (A) Buried group; (B) Surface group; Figure S4: Model error under different hyperparameters. (A) Skin samples of the buried group; (B) Soil samples of the buried group; (C) Skin samples of the surface group; (D) Soil samples of the surface group; Table S1: Soil parameters table; Table S2: Results of PERMANOVA testing the effects of PMI, type and their interaction on beta diversity.
