# Supplementary figures and images for: Estimating Postmortem Interval of Buried Pig Carcasses by Integrating Microbial Succession Patterns with Machine Learning Algorithms
Source: Microorganisms. 2025 Dec 19;14(1):6. doi: 10.3390/microorganisms14010006 (PMC12844239; doi:10.3390/microorganisms14010006)

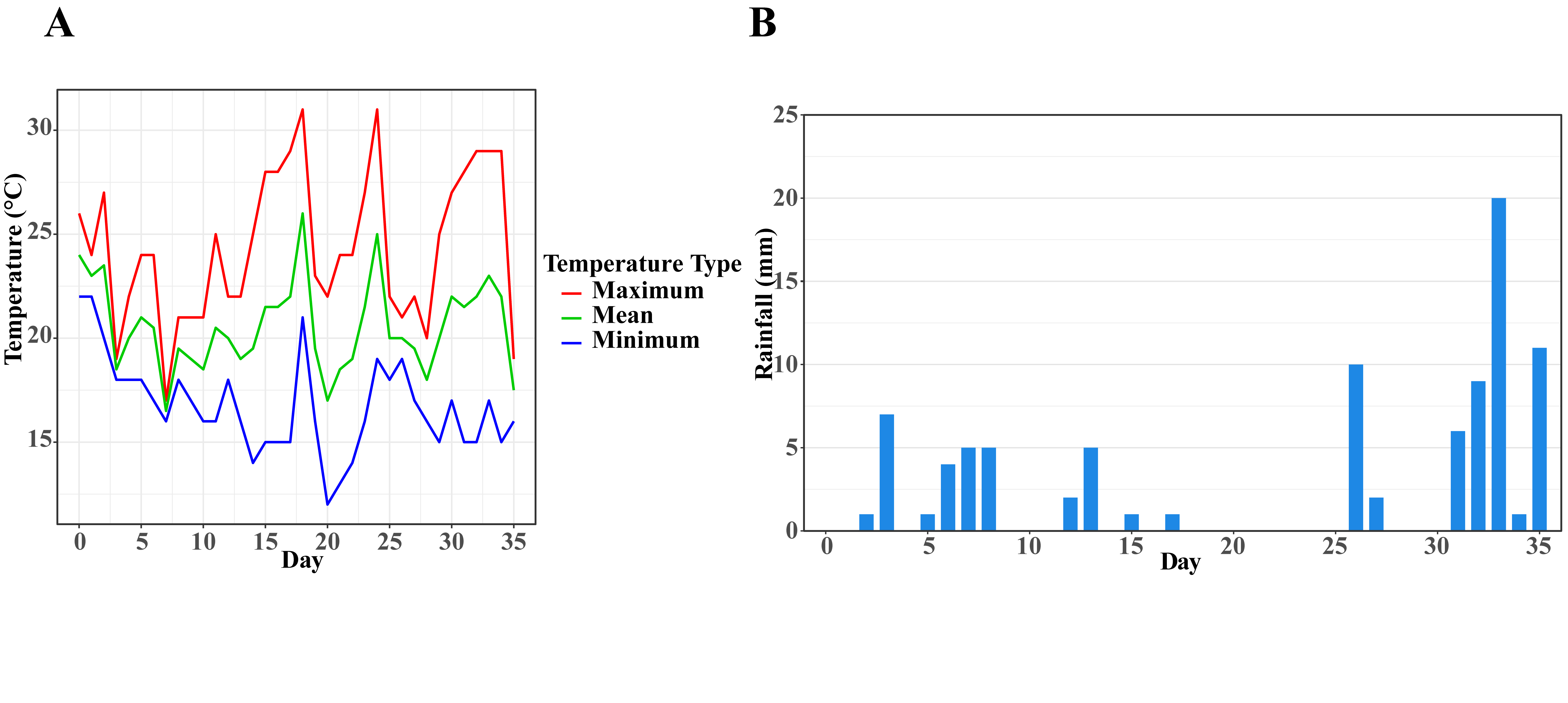

Supplement: Supplementary file 1 [file microorganisms-14-00006-s001.zip › Figure S1.tif]

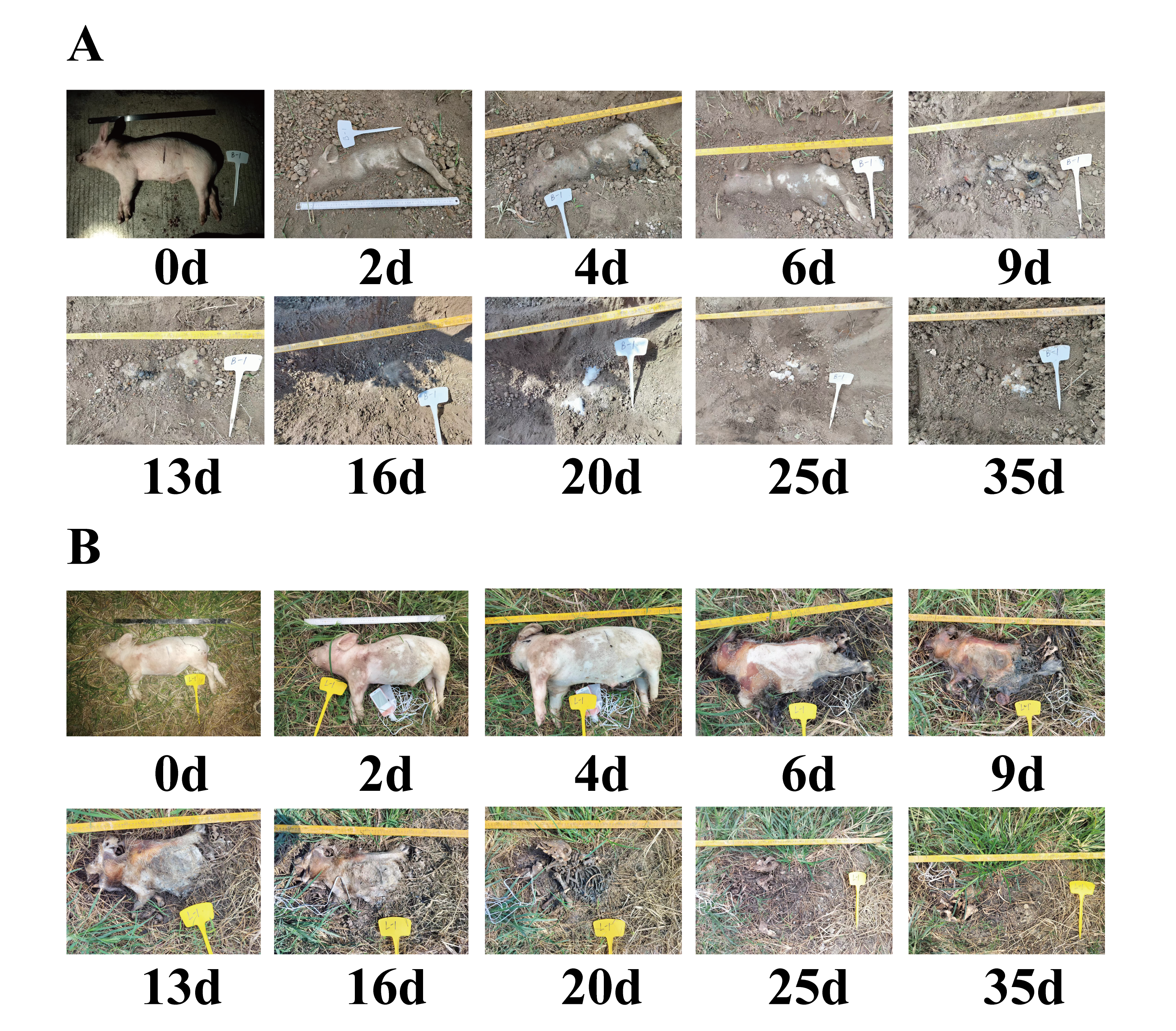

Supplement: Supplementary file 1 [file microorganisms-14-00006-s001.zip › Figure S2.tif]

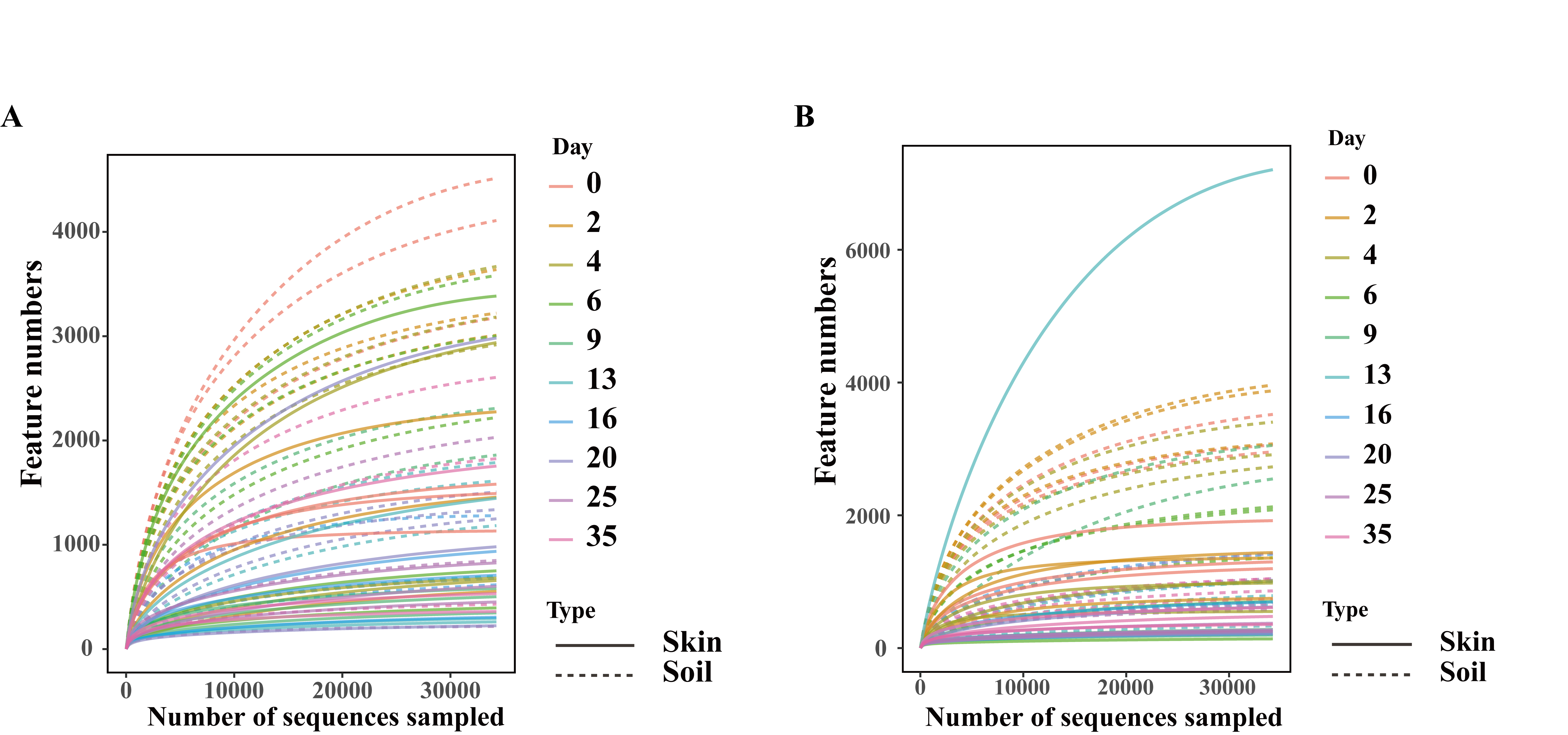

Supplement: Supplementary file 1 [file microorganisms-14-00006-s001.zip › Figure S3.tif]

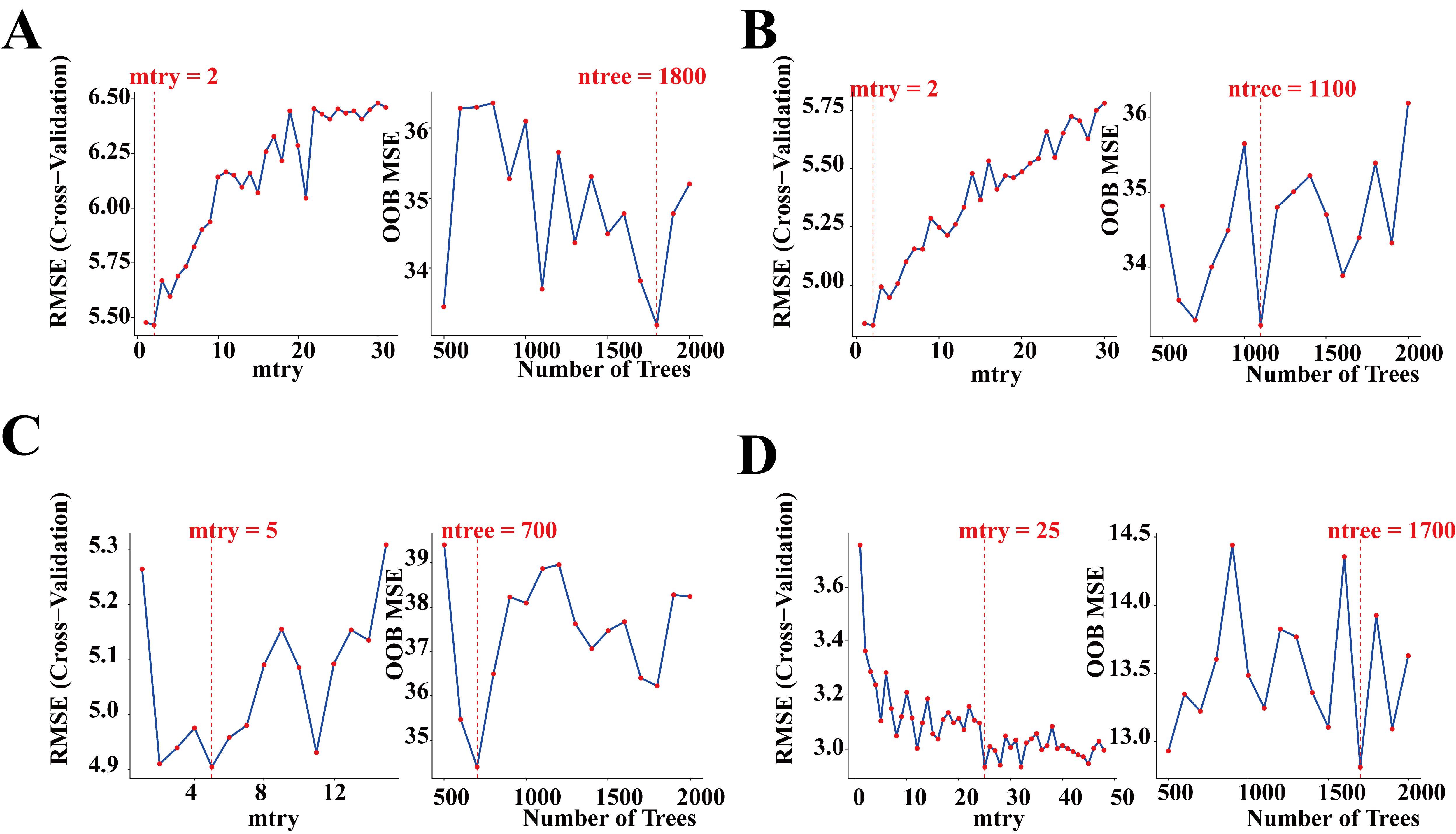

Supplement: Supplementary file 1 [file microorganisms-14-00006-s001.zip › Figure S4.tif]
